# Drugs incorporated into the Brazilian Unified Health System: document analysis of methodological decisions in budget impact analyses between 2012 and 2024

**DOI:** 10.1590/S2237-96222025v34e20250122.en

**Published:** 2025-09-08

**Authors:** Daniel da Silva Pereira Curado, Everton Nunes da Silva

**Affiliations:** 1 Universidade de Brasília, Programa de Pós-Graduação em Saúde Coletiva, Brasília, DF, Brazil; 2 Ministério da Saúde, Secretaria-Executiva da Comissão Nacional de Incorporação de Tecnologias no Sistema Único de Saúde, Brasília, DF, Brazil; 3 Universidade de Brasília, Faculdade de Ciências e Tecnologias em Saúde, Brasília, DF, Brazil

**Keywords:** Budget Impact Analyses, Health Technology Analysis, Drugs, Unified Health System, Document Analysis, Análisis de Impacto Presupuestario, Evaluación de Tecnologías Sanitarias, Medicamentos, Sistema Único de Salud, Análisis de Documentos

## Abstract

**Objective:**

Systematize the methodological decisions adopted in the budget impact analyses of the recommendation reports of the National Commission for the Incorporation of Technologies into the Unified Health System (Conitec) regarding drugs incorporated into the SUS (Brazilian Unified Health System) in the period from 2012 to 2024.

**Methods:**

This is an exploratory, descriptive, retrospective study, based on document analysis of Conitec’s technical recommendation reports with decisions on the incorporation of drugs published up to 2024. Information from the Budget Impact Analyses (BIA) was extracted and presented in terms of percentage, median and interquartile range.

**Results:**

291 analyses for the incorporation of drugs were identified. More than half of these were requested by the public sector (65.3%). Chronic non-communicable diseases were most frequent (39.2%), followed by infectious diseases (22.3%) and rare conditions (21.0%). The majority of the drugs included represented new additions (55.3%). The presence of BIA in the analyses was quite gradual, reaching 100% only from 2019 onwards. 64.5% used claims data-based and 84.0% conducted sensitivity analyses in the BIAs analysed. The total median of the incorporation diffusion (market share) ranged from 30.0% (1st year) to 72.0% (5th year). The cost analyses were essentially focused on the acquisition cost of drugs. However, the quantity of pharmaceutical units was not clearly reported in 55.5% of the analyses.

**Conclusion:**

The findings indicate that methodological inconsistencies persist in BIAs, such as the absence of sensitivity analyses and cost analyses limited to the acquisition cost of drugs. Therefore, it is suggested that there may be weaknesses in the estimates of the real budget impacts of technologies on the SUS.

## Resumen

**Objetivo:**

Sistematizar las decisiones metodológicas adoptadas en los estudios de impacto presupuestario de los informes de recomendaciones de la Comisión Nacional para la Incorporación de Tecnologías al Sistema Único de Salud (Conitec) sobre medicamentos incorporados al SUS en el período de 2012 a 2024.

**Métodos:**

Se trata de un estudio exploratorio, descriptivo, retrospectivo, mediante análisis documental de informes de recomendaciones técnicas de Conitec con decisiones sobre la incorporación de medicamentos publicados hasta 2024. Se extrajo información del Análisis de Impacto Presupuestario (AIP) y se presentó por porcentaje, mediana y rango intercuartil.

**Resultados:**

Se identificaron 291 análisis para incorporación de fármacos. Más de la mitad de éstas fueron solicitadas por el sector público (65,3%). Las enfermedades crónicas no transmisibles fueron las más frecuentes (39,2%), seguidas de las infecciosas (22,3%) y las raras (21,0%). La mayoría de los medicamentos incluidos representaron nuevas incorporaciones (55,3%). La presencia de AIP en los análisis fue bastante gradual, llegando al 100% recién a partir de 2019. Hubo una adopción del 64,5% de la demanda medida y del 84,0% de análisis de sensibilidad en el total de AIP. La mediana total de difusión de la incorporación (cuota de mercado) varió del 30,0% (primer año) al 72,0% (quinto año). Los análisis de costos se centraron esencialmente en el coste de adquisición de los medicamentos. Sin embargo, en el 55,5% de los análisis no se informó claramente la cantidad de unidades farmacéuticas.

**Conclusión:**

Los hallazgos indican que persisten inconsistencias metodológicas en las AIP, como la ausencia de análisis de sensibilidad y análisis de costos limitados a los valores de adquisición de medicamentos. Por tanto, se sugiere que pueden existir debilidades en las estimaciones de los impactos presupuestarios reales de las tecnologías en el SUS.

**Ethical aspects**

This research used public domain data and anonymized databases.

## Introduction

The adoption of Health Technology Assessment (HTA) has been increasing worldwide, especially in universal health systems. In this context, a comprehensive economic assessment is essential to inform decision-making on the potential incorporation of a technology (1,2).

Given that a technology may be considered cost-effective but not affordability in terms of population, Budget Impact Analyses (BIA) are essential to complement Cost-Effectiveness Analysis (CEA) (3-5). In the case of CEA, a measure of allocative efficiency is provided by weighing costs and clinical benefits. BIA, in turn, estimates the impact on health financial resources in the short/medium term, which informs budgetary viability (affordability) to decision makers (1,6,7). 

Methodological guidelines for the preparation of the BIA have been established globally, including Brazil (8). In the Brazilian guidelines, recommendations are made on several methodological steps, including the disease, new treatment under evaluation and comparators, analysis perspective, time horizon, target population estimate, use of resources (costs), diffusion and speed of incorporation (market share) and proposed scenarios (9). However, weaknesses in BIA conduction continue to be observed worldwide (10) and nationally (11,12).

In general, publications on reports from the National Commission for the Incorporation of Technologies into the Unified Health System (Conitec) are limited to the presence or absence of BIA in the incorporation proposals (11,13,14). It is therefore necessary to delve deeper into the methodological characteristics of these studies and go beyond the information on recommendations for the incorporation of drugs into the Unified Health System (SUS) (13). This provides a general and current overview of the application of the recommendations of the Brazilian BIA guidelines, as well as the identification of any improvements over the years.

The objective of the study is to systematize the methodological decisions adopted in the budget impact analyses of Conitec’s recommendation reports regarding drugs incorporated into the SUS from 2012 to 2024.

## Methods

### Study context

Conitec was created by Law No. 12.401/2011, to advise the Ministry of Health on the incorporation of health technologies into the SUS (15,16). Thus, there was a legal institutionalization of HTA in the country (17). According to Decree No. 7,646/2011, proposals submitted to Conitec must include BIA for the technologies evaluated, among other requirements. The technical reports support Conitec’s recommendations, which consider: i) clinical evidence of the technology; ii) comparative economic evaluation of benefits and costs; iii) budget impact of incorporating the technology into the SUS. The main recommendations on BIA preparation are summarized in Table 1.

**Table 1:** Methodological steps and main recommendations of the methodological guidelines for budget impact analysis

Budget Impact Analysis Definitions	Main recommendations
**Population eligible for the proposed technology**	There is a need to: i) establish exactly what the indication for the new treatment will be in the health system, prioritizing patients who will have the greatest therapeutic benefit and/or allocative efficiency, according to the Cost-Effectiveness Analysis (CEA); ii) estimate the proportion of patients who fit the established indication.
**Proposed technology**	It must contemplate the technology being proposed.
**Analysis perspective**	Definition of one of the following perspectives: SUS (Brazilian Unified Health System), in its different spheres; Supplementary health system (private health plans and insurance); Local perspectives on the health system, such as hospitals and health centers.
**Time horizon**	Period of one to five years, with budget impact estimates reported year by year. Longer period for technologies whose market demand stabilization requires more time.
**Population approach**	Epidemiological method: data on the prevalence and incidence of the clinical condition of interest, specifically considering its use of the health system adopted in the perspective; Claims data-based method: i) count of registered patients, when there is some type of specific registration system or; ii) historical number, or for a given year, of reimbursement requests for medical treatment in a specific health plan.
**Reference scenario**	Current pattern of use of available treatments for the clinical condition from the adopted perspective. Current treatments should be described considering the same items used for the new intervention.
**Alternative scenarios**	It is recommended that one to three alternative scenarios be simulated. Each alternative scenario should be designed based on projections of market shares and the rate of incorporation of the technology evaluated.
**Market share of the reference scenario**	Information from official bodies or scientific publications on the national market is preferred. Alternatively, estimates of market shares (current and projected) from other countries may be considered. The use of an expert panel (through the Delphi method, for example) can be used to characterize the current market when other data sources are not available.
**Market share of alternative scenarios**	The following factors should be included in the projection of diffusion rates: the availability of equipment, training and qualification of personnel, the operation of the entire system, and the habits of doctors and patients. It should be considered that some therapies are quickly incorporated, due to their facilities, benefits and market pressure, while others have their incorporation delayed, and may not reach 100%.
**Treatment costs**	All costs considered must reflect the perspective of the health budget manager for whom the study is intended, that is, the amount that will actually be disbursed by the manager after the incorporation in question. Only direct costs should be considered, which include the costs of the new technology itself and those directly associated with its use, such as those related to hospitalizations for application, adjuvant interventions and drugs used to prevent or treat adverse events. This combined cost “therapeutic package” must be calculated for the proposed new technology, in its different alternative scenarios, and for the other interventions that make up the reference scenario.
**Avoided costs**	Costs to be avoided as a result of a clinical benefit of the new technology can be estimated, such as by reducing the need for hospitalizations, surgeries, hemodialysis or certain drugs.
**Inflation and discount rate**	These adjustments are not recommended due to the short time horizon and the fact that the BIA amount corresponds to a present value used in the manager’s budget estimates.
**Sensitivity analysis**	Due to its practicality in execution and easy interpretation of results, sensitivity analysis by scenarios is recommended.

Content adapted from the BIA Methodological Guidelines (9).

### Study design 

This is an exploratory, descriptive, retrospective study, based on document analysis of Conitec’s technical recommendation reports on the incorporation of drugs between 2012 and 2024. The analysis unit were Conitec’s reports on drugs incorporated into the SUS. From these, information was extracted about BIAs and variables that characterize the demands. Because they are secondary data, the RECORD tool (18) was used to guide the study report.

Technical reports were selected because these documents record the procedural process for incorporation into the SUS, supporting the decision-making by the Ministry of Health. Furthermore, demands initiated by the public sector have their BIAs publicly presented only in their respective technical reports, as well as recalculations made by reviewers in critical analyses of demands from the private sector. 

The technologies of interest in this study were drugs, because they represent a large part of the demands for incorporation into the SUS. They are also one of the items with the greatest impact on the family budget of Brazilians (19), especially in low-income households (20). Furthermore, surveys up to 2016 showed that most of Conitec’s analyses referred to drugs (11,13). In fact, this pattern has become more pronounced in recent years, according to the monitoring panel on the Conitec website, which points to a significant predominance of drugs in the process of incorporation into the SUS.

### Sample 

The inclusion criteria were: i) this is a demand for incorporation of the drug; ii) it had a recommendation for incorporation or expansion of use in the SUS; and iii) it had a decision published between January 1, 2012, and December 31, 2024. The exclusion criteria was: i) this is a re-evaluation of a drug that did not change the previous decision.

### Variables and data sources

The data source was the Conitec website, where all recommendation reports for the technologies analyzed are publicly available (https://www.gov.br/conitec/pt-br/assuntos/avaliacao-de-tecnologias-em-saude/recomendacoes-conitec). When there were concerns about the reporting of reports on private sector demands, the applicants’ files (available only from 2019 onwards) were additionally consulted, which remain public during the public consultation period on the topic. When necessary, recordings of Conitec meetings were also checked (available only from 2020 onwards).

Data extracted were the name of the drug; clinical condition; applicant, year of the incorporation decision; type of population approach; quantity of eligible population in the 5th year; percentages of market share in the 1st and 5th years; number of active comparators; and cost components considered in BIA. The constructed database, with the formulas used, is available in the Mendeley Data repository (21). The variables adopted and their descriptions are detailed in Table 2.

**Table 2:** Variables defined for categorizing demands for drugs incorporated into the Unified Health System

Variable	Description
**Type of applicant**	Categorical variable with the classifications below.
Public	Incorporation proposals requested only by the Ministry of Health and other public administration bodies or by both the public sector and private institutions.
Private	Incorporation proposals requested exclusively by private institutions.
**Type of incorporation**	Categorical variable with the classifications below and defined by consulting the National List of Essential Drugs (Relação Nacional de Medicamentos Essenciais, Rename) and the reports of the National Commission for the Incorporation of Technologies into the Unified Health System (Conitec).
New incorporation	A drug without clear official registration of previous incorporation for the clinical condition under analysis or for another condition in the SUS.
Expansion of use	A drug already incorporated for another disease or for the same disease, but in another subgroup in the SUS. For example, severe (1st incorporation) and moderate (2nd incorporation) illness.
New presentation	A drug already incorporated for the indication under analysis, but in a different pharmaceutical presentation from that proposed. For example, a vial (intravenous) already present in the SUS and the inclusion of a tablet (oral) for the same therapeutic indication was evaluated.
**Type of clinical condition**	Categorical variable with the classifications below and defined based on the description present in each technical report. In case of doubt, the available Clinical Protocols and Therapeutic Guidelines (Protocolos Clínicos e Diretrizes Terapêuticas, PCDT) were consulted. The specific clinical condition in which the drug would act was considered. For example, antibiotics for a certain bacterial infection in a subgroup of patients with a rare disease would lead to classification as an infectious disease.
Preventable	Clinical conditions that can be prevented through prophylactic drugs, such as vaccines and contraceptives.
Infectious	Infectious diseases, except those covered under preventable.
Rare	Diseases with a prevalence of up to 65 people in every 100,000 individuals, according to the National Policy for Comprehensive Care for People with Rare Diseases of the Ministry of Health (Ordinance GM/MS No. 199/2014). If necessary, the Orphanet database (https://www.orpha.net/), dedicated to rare diseases, was consulted.
Oncological	Diseases classified as cancers and syndromes treated in oncology.
Chronic non-communicable disease –CNCD	Chronic non-communicable clinical conditions, except those covered under rare and oncological conditions.
**Presence of Budget Impact Analysis** (BIA)	Dichotomous variable to indicate what was considered a minimally conducted or reported BIA. The presence of A BIA was defined as those reports that presented, at the very least, the budget impact amounts during the adopted time horizon.
**Type of population approach**	Categorical variable with the classifications below.
Epidemiological	Approach described in the report as exclusively epidemiological, such as the use of prevalence and incidence data from publications or population estimates.
Claims data-based	Adoption of measured demand data, together with epidemiological data or not. To define measured demand, the express description in the report or the citation on the use of DATASUS databases or national records of data collected in that population was considered.
**No. of active comparators**	Quantity of pharmacologically active comparators (other drugs) considered in BIA, in a distinct manner. That is, without being grouped into one treatment proxy.
**Absence of therapeutic alternative**	Dichotomous variable on the non-use of one (or more) pharmacologically active comparator (another drug) with a specific indication for the clinical condition in BIA.
**Estimated population**	Number of people in the population eligible for the proposed drug in the last year of the time horizon. Thus, it differs from the population in use, as it was the total number of people without any stratum, such as the application of percentages of market share.
**Market share**	Percentages of diffusion of the proposed drug in the 1st and 5th year of BIA, with variations between 0% and 100%, defined in the report.
**Sensitivity analysis**	Dichotomous variable on carrying out sensitivity analysis, using a tornado diagram or scenario analysis. For this, the presentation of at least one additional scenario was considered.
**Cost components**	Categorical variable, defined as “yes”, “not specified” or “no”, and subdivided into “identification”, “measurement” and “valuation”. The first refers to the mention of adoption of the cost component in the analysis. The second refers to the report of the specific quantity of units considered (such as x tablets per year). And the last one was the presentation of the monetary amount attributed to the unit of the cost component (such as the price of the drug).
Acquisition cost of drugs	Costs related exclusively to the acquisition of drugs. For measurement, it was considered as “not specified” if the report only reported the dosage used.
Costs of the drug administration	Costs related to the explicit procedure of drug administration. In the measurement, it was considered that the quantity was implicit if the annual quantity of use of the drug was informed.
Costs of consultations/visits by healthcare professionals	Costs of clinical care provided by physicians and other healthcare professionals, including hospitalization/admission and other clinical procedures conducted by professionals that were countable and described in the report.
Monitoring and follow-up exams	Costs of clinical or laboratory tests/examinations described as monitoring procedures during the treatment in which the proposed drug will be used.
Management of adverse events	Costs of procedures with the exclusive purpose of identifying and managing adverse events, which may include laboratory tests of specific biochemical indicators and the use of drugs to manage undesirable effects of the proposed drug.
Other	Costs related to various less frequent procedures not previously covered, such as a specific surgery, transplant or diagnostic test prior to treatment.

It is worth noting that the classification of the public sector demands (Table 2) included those also coming from private institutions because, in reports that also had the public administration as an applicant, Conitec members often considered the calculations conducted by the reviewers to be more appropriate, within the scope of the so-called internal demand.

Although a large part of preventable conditions are infectious diseases and rare and oncological diseases are also chronic non-communicable diseases (CNCD), this disaggregation was chosen to cover the specificities of these conditions and their drugs. Prophylactic technologies (mainly vaccines and contraceptives) commonly have different dosing schedules and have a larger eligible population. This is because these people are still susceptible to the disease to be prevented, which sets a different purpose than the treatment of infectious diseases such as HIV infection or hepatitis C. In the case of CNCD, rare diseases have a smaller population size, but a greater demand for therapeutic alternatives, which is often reflected in larger percentages of market share. Oncological diseases, in turn, are not only accompanied by considerable costs, but also by a drug financing policy that is different from that adopted for CNCDs in general (oncology policy and pharmaceutical assistance policy, respectively).

The population approach adopted in BIA, in turn, had the classification of claims data-based grouped (Table 2) to refer to analyses that were essentially based on measured demand data, even if they partially used epidemiological data. That is, when the population base for the calculation was the current number of SUS users, for example. This methodological choice was made to better reflect the magnitude of the population estimated in each of the approaches, commonly being numerically superior in the epidemiological one (9). Thus, BIAs that used both the epidemiological approach and claims data-based tend to obtain estimates closer to BIAs supported only by claims data-based than to those with the epidemiological approach exclusively.

Comparators mentioning “standard treatment”, “supportive care”, “palliative care” or related terms that did not explicitly include a drug with specific action in that disease were considered to have “absence of active comparator”. Furthermore, only active comparators that were clearly presented in BIA (e.g. with market share and/or distinct values) were accounted for. If a drug proxy was used, only one active comparator was counted.

Due to the presentation of different scenarios in BIAs, the one that was most representative of the incorporation was defined, from which the data was taken. To this end, the following criteria were considered, in the following order: i) final recommendation from Conitec, defining the population and subgroups covered; ii) recalculation after new information, such as requests from Conitec for adjustments or new price proposals sent by companies; iii) explicit position of the reviewers on the most appropriate scenario, presented to Conitec as the main result; iv) more gradual diffusion, indicating more feasible implementation in SUS; v) use of claims data-based data, which tends to be more reliable for the eligible population. It should be noted that, in cases where more than one drug was evaluated in the same report, scenarios were selected in which the incorporated drugs were considered in a complementary manner. When BIA information was not disaggregated, it was classified as a single BIA, regardless of the quantity of drugs incorporated. 

Both the number of eligible population and the market share percentages were directly extracted from the drug report. However, when there was an issue of missing data in a report, we sought to obtain the information by calculating the remaining data, when possible. For example, the number of patients using the drug and the market share in the 5th year were sufficient to obtain the eligible population in the 5th year.

As for the cost component, the focus was on the clarity of the description of each item, in terms of identification (listing the items), measurement (quantifying) and valuation (assigning value) (22). In this sense, with the exception of the drug administration measurement, no inference was made about the quantity of units in BIA. However, “not specified” was used when the quantity of a component was not present, despite its inclusion. This classification was also given when the report had evidence of that cost component, but did not detail it.

### Statistical analysis

The data were analyzed in an aggregated form, being presented by percentage, median and interquartile range to represent measures of central tendency and dispersion, respectively. 

To avoid potential sources of bias (18), all reports with available BIA data were considered. Furthermore, possible interpolation and recalculation of missing data due to reporting errors were performed.

Subgroup analyses were performed to demonstrate differences between drugs. These stratifications were made by year of incorporation and type of clinical condition. The results are shown in tables, with the appropriate stratifications and totals.

## Results

During the period, 291 analyses for the incorporation of drugs were identified. More than half of these were requested by the public sector (65.3%), ranging from 44.4% in 2018 to 92.3% in 2014 and 2015 over the period. CNCDs were most frequent (39.2%), followed by infectious (22.3%) and rare (21.0%) diseases. However, this pattern was heterogeneous during the years evaluated, without a well-defined trend for the clinical condition stratified by year (Table 3).

**Table 3:** General characterization of the demands for drugs incorporated into the Unified Health System between 2012 and 2024. Brazil, 2022 (n=291)

Year (n)	Applicant	Clinical condition					Type of incorporation		Presence of Budget Impact Analysis n (%)
	Public n (%)	Preventable n (%)	Infectious n (%)	Rare n (%)	Oncological n (%)	Chronic non-communicable disease n (%)	New incorporation n (%)	Expansion of use n (%)	
**2012** (24)	22 (91.7)	1 (4.2)	3 (12.5)	3 (12.5)	2 (8.3)	15 (62.5)	20 (83.3)	4 (16.7)	-
**2013** (15)	12 (80.0)	4 (26.7)	1 (6.7)	3 (20.0)	4 (26.7)	3 (20.0)	12 (80.0)	3 (20.0)	2 (13.3)
**2014** (13)	12 (92.3)	-	2 (15.4)	2 (15.4)	5 (38.5)	4 (30.8)	2 (15.4)	10 (76.9)	3 (23.1)
**2015** (26)	24 (92.3)	-	12 (46.2)	-	1 (3.8)	13 (50.0)	6 (23.1)	14 (53.8)	5 (19.2)
**2016** (12)	7 (58.3)	-	3 (25.0)	-	-	9 (75.0)	3 (25.0)	8 (66.7)	6 (50.0)
**2017** (24)	13 (54.2)	-	6 (25.0)	9 (37.5)	1 (4.2)	8 (33.3)	11 (45.8)	10 (41.7)	16 (66.7)
**2018** (27)	12 (44.4)	-	10 (37.0)	10 (37.0)	2 (7.4)	5 (18.5)	19 (70.4)	4 (14.8)	22 (81.5)
**2019** (19)	11 (57.9)	2 (10.5)	2 (10.5)	3 (15.8)	2 (10.5)	10 (52.6)	11 (57.9)	4 (21.1)	19 (100.0)
**2020** (25)	14 (56.0)	2 (8.0)	6 (24.0)	5 (20.0)	4 (16.0)	8 (32.0)	15 (60.0)	7 (28.0)	22 (88.0)
**2021** (19)	10 (52.6)	2 (10.5)	2 (10,5)	4 (21.1)	3 (15.8)	8 (42.1)	12 (63.2)	3 (15.8)	19 (100.0)
**2022** (35)	20 (57.1)	2 (5.7)	9 (25.7)	10 (28.6)	3 (8.6)	11 (31.4)	20 (57.1)	13 (37.1)	34 (97.1)
**2023** (22)	11 (50.0)	2 (9.1)	4 (18.2)	7 (31.8)	2 (9.1)	7 (31.8)	14 (63.6)	3 (13.6)	22 (100.0)
**2024** (30)	22 (73.3)	-	5 (16.7)	5 (16.7)	7 (23.3)	13 (43.3)	16 (53.3)	14 (46.7)	30 (100.0)
**Total** (291)	**190** (65.3)	**15** (5.2)	**65** (22.3)	**61** (21.0)	**36** (12.4)	**114** (39.2)	**161** (55.3)	**97** (33.3)	**200** (68.7)

The color gradient in each column represents the range of findings. Therefore, darker shades of color indicate higher percentages in the respective column.

Most of the drugs included represented new incorporations (55.3%), with percentages above 53.0% during the period, except from 2014 to 2017, when expansions in use stood out. On the other hand, the incorporation of new presentations has always been quite discreet, never exceeding 24.0%, the percentage for 2015 (Table 3).

As observed in Table 3, the presence of BIA in technical reports on drugs was quite gradual in the first years of Conitec, reaching 50.0% in 2016. As of 2018, BIA percentages remained above 80.0%. With the exception of 2020 and 2022, in which percentages of 88.0% and 97.1% were obtained, BIA was adequately contemplated in all analyses conducted in recent years.

Regarding the methodological choices of BIAs (Table 4), the analyses of drugs for preventable conditions stood out, as they had a larger estimated population than those for other clinical conditions. This pattern was also noticed for market share in the 1st and 5th year regarding preventable diseases (medians of 75.0% to 100.0%, respectively). In second place, in terms of estimated population size, were CNCDs. However, CNCDs presented the lowest percentages of market share (medians of 26.0% in the 1st year and 60.0% in the 5th year). In terms of population and market share, rare diseases presented a profile quite similar to oncological diseases (relatively small population and moderate market share).

**Table 4:** Methodological characteristics adopted in the budget impact analyses relating to drugs incorporated into the Unified Health System between 2012 and 2024. Brazil, 2025 (n=291)

Type of clinical condition	Estimated population (n; IQT)^a^	Market share (%; IQT)^a^	Comparators (n; IQT)^a^	No therapeutic alternative n (%)	Claims data-based n (%)	Sensitivity analysis n (%)
Preventable	1,887,115 (270,914; 5,323,049)	**1st year**: 75.0 (19.3; 100.0)	1 (0; 1)	4 (44.4)	4 (44.4)	8 (88.9)
		**5th year**: 100.0 (21.3; 100.0)				
Infectious	3,115 (592; 41,114)	**1st year**: 50.0 (30.0; 100.0)	1 (1; 1)	8 (20.5)	23 (58.9)	33 (84.6)
		**5th year**: 95.0 (50.0; 100.0)				
Rare	1,550 (232; 5,296)	**1st year**: 50.0 (20.0; 65.0)	1 (0; 2)	22 (44.9)	29 (59.2)	40 (81.6)
		**5th year**: 80.0 (45.0; 95.0)				
Oncological	737 (224; 3,849)	**1st year**: 30.0 (10.0; 87.5)	1 (1; 1)	4 (14.8)	20 (74.1)	20 (74.1)
		**5th year**: 70.0 (50.0; 100.0)				
**Chronic non-communicable diseases**	28,150 (9,087; 85,747)	**1st year**: 26.0 (10.0; 50.0)	1 (1; 2)	15 (19.7)	53 (69.7)	67 (88.2)
		**5th year**: 60.0 (36.2; 100.0)				
Total	5,619 (814; 45,228)	**1st year**: 30.0 (15.0; 100.0)	1 (0; 1)	53 (26.5)	129 (64.5)	168 (84.0)
		**5th year**: 72.0 (45.0; 100.0)				

IQT – interquartile range; ^a^Median and IQT (1st and 3rd quartiles, respectively).

As for the absence of a therapeutic alternative, that is, a pharmacologically active comparator in BIA, rare and preventable diseases presented percentages close to 45.0%, while other diseases did not exceed 21.0%. However, the various analyses without therapeutic alternatives between preventable (n=4) and rare (n=22) conditions stand out (Table 4).

Oncological diseases represented the type of clinical condition in which claims data-based in the population approach was most frequently adopted, accounting for 74.1% of BIAs. In any case, the vast majority of clinical conditions demonstrated percentages above 58.0%, except for preventable diseases (44.4%) (Table 4).

Regarding sensitivity analyses, preventable conditions and CNCDs stood out with percentages above 88.0%. While oncological diseases presented a lower percentage (74.1%), the conduction of sensitivity analysis in general for BIAs was 84.0% (Table 4).

Table 5 shows that drugs were the cost component most covered in BIAs, being present in all analyses. However, it is worth noting that, in more than half of the cases (55.5%), the number of units was not clearly reported. Also, this measurement domain presented the lowest percentages in all cost components. However, the management of adverse events and other events were the least covered items in all domains in BIAs (≈16.0%).

**Table 5:** Cost components considered in budget impact analyses relating to drugs incorporated into the Unified Health System between 2012 and 2024. Brasil, 2025 (n=200)

Cost component	Identification		Measurement		Valuation	
	Yes n (%)	Not specified n (%)	Yes n (%)	Not specified n (%)	Yes n (%)	Not specified n (%)
Drug	200 (100.0)	0 (0.0)	89 (44.5)	111 (55.5)	198 (99.0)	1 (0.5)
**Drug administration**	31 (15.5)	0 (0.0)	16 (8.0)	15 (7.5)	28 (14.0)	3 (1.5)
**Consultations/visits by healthcare professionals**	51 (25.5)	1 (0.5)	19 (9.5)	33 (16.5)	49 (24.5)	3 (1.5)
**Monitoring and follow-up exams**	46 (23.0)	1 (0.5)	21 (10.5)	26 (13.0)	43 (21.5)	4 (2.0)
**Management of adverse events**	31 (15.5)	0 (0.0)	9 (4.5)	21 (10.5)	28 (14.0)	2 (1.0)
Other	32 (16.0)	0 (0.0)	8 (4.0)	24 (12.0)	27 (13.5)	5 (2.5)

In each column, the range of findings is represented by the gradient of the colors green (higher percentages), yellow (median percentages) and red (lower percentages). Thus, darker shades of each color indicate values that tend towards the extremes (green and red) or central point (yellow) in the respective column.

## Discussion

There has been methodological progress in BIA of drugs incorporated into SUS, especially since 2019. However, inconsistencies were observed in the application of BIA guidelines, especially regarding the cost components included. Most of the analyses considered only the costs of the drugs under investigation, not including other costs related to the treatment of the health condition. Furthermore, no sensitivity analyses were carried out on part of the incorporation reports, which implies the absence of an assessment of uncertainties. These findings indicate that there may be weaknesses in the estimates of the real budgetary impacts of technologies on SUS.

This was the first study to systematize BIA characteristics of Conitec reports, comparing them to the BIA methodological guidelines. To this end, clear and objective definitions were proposed. Furthermore, exclusively public and official data from Conitec were used. This allows for the reproducibility and continuity of monitoring of BIAs for drugs incorporated into SUS. However, some limitations present in this study should be highlighted. First, some information relevant to understanding BIAs was incomplete or missing in the Conitec reports, which reduced the sample for our analysis and did not represent all BIAs for the incorporation of drugs in SUS. Secondly, the definition of “presence of a BIA” implied that certain reports with insufficient BIA information were not included. It is justified, however, that the incompleteness of these reports would prevent such data from being added to the results of this study and would potentially increase the heterogeneity of the findings. Thirdly, the first year of BIAs does not necessarily correspond to the year of incorporation of the drug, which may represent distortions in the estimated population. Fourthly, the drugs and their comparators that were not disaggregated in BIAs were considered by us as just one drug, which does not represent the reality of clinical practice. Fifthly, the information used was restricted to just one of the scenarios described in the drug report, selected by the criteria we defined. This scenario does not necessarily represent the reality of drugs in SUS, which will potentially involve several variables that we are unaware of. Sixthly, some missing BIA information was calculated by us when the other information reported allowed this procedure. The reason for this is that in this way we sought to mitigate data loss in the analyses. However, we recognize the possibility of inconsistencies in these calculated data, despite them being based exclusively on the other information described in the reports.

The current version of BIA methodological guidelines, published in 2012, has been made available to guide the development of the BIA (9). With the legal requirement of compliance with the methodological guidelines to propose incorporations into SUS, the applicants had to adapt themselves, regarding the presentation of this type of study. A survey carried out between 2012 and 2015 revealed that 17.3% of the submissions were not assessed by Conitec because they were rejected in the prior documentary analysis due to the absence of a specific BIA (23). This percentage is close to the average presence of BIA between 2012 and 2015 observed in our study (13.9%). It is worth noting that this was the period in which the incorporated drugs were mostly demanded by the public sector, which may be related to the assessment by Conitec even without compliant BIAs.

Gradually, applicants adapted their work to the BIA guidelines, reaching 100% in 2019. However, in 2020 and 2022, these percentages fell in some public sector proposals for the inclusion of new pharmaceutical presentations – drugs already available in SUS, but in different presentations from those proposed. These cases reinforce the discussion about the relevance of incorporating new pharmaceutical presentations into SUS, within the scope of Conitec, when there are no relevant concerns about their efficacy/effectiveness, safety, cost-effectiveness and budget impact. Furthermore, suggestions have been made to “streamline the process of submitting technology assessment proposals to Conitec, when submitted by SUS entities” in discussions promoted by the Ministry of Health (24).

According to BIA guidelines, the epidemiological method for estimating the eligible population tends to overestimate the actual number of people (9). Thus, the guidelines’ recommendation to give preference to estimates with claims data-based may explain the majority adoption of measured demand in the BIAs we analyzed. Additionally, smaller populations, obtained through claims data-based, result in smaller budget impact values. As an important element in the decision-making process (6), BIA can be decisive regarding the incorporation of the drug. Until 2016, 25.8% of the drugs incorporated into SUS had a low financial/budget impact as justification in the Conitec report (13).

Given that real-world data are not always available at the time of drug incorporation, initiatives have been implemented by the Ministry of Health to mitigate risks to the sustainability of SUS. In 2017, methodological guidelines for evaluating the performance of health technologies focusing on real-world data were published (25). Subsequently, Ordinance GM/MS No. 4.228/2022 allowed Conitec’s deliberations to be provisional and subject to the reassessment of parameters, including the budget impact of technology on SUS. Measures such as these facilitate the review of incorporations after significant differences are identified between the estimated and actual budget impact (12).

The high adoption of claims data-based observed in this study may be related to the general percentages of market share. This would include the recommendation of the BIA guidelines to consider drug dispensing or reimbursement data as one of the ways to define the market share (9). It is worth noting that the lowest percentages correspond to drugs incorporated for CNCDs, which mostly require the preparation or updating of a PCDT (Clinical Protocol and Therapeutic Guidelines) for the disease to be made available in SUS. This PCDT process, acquisition and dispensing of drugs for prevalent conditions, commonly requires several months after the incorporation itself (26), which can be considered in the definition of market share. Furthermore, the majority of demands referred to CNCDs, which had one of the lowest absences of therapeutic alternatives (19.7%). This indicates that many of these drugs were incorporated as yet another therapeutic option for that clinical condition and, consequently, began to share the market with them. Such a fact would justify their lower market share, which does not reach 100% after five years of BIA.

Drugs incorporated for oncological diseases, in turn, presented a population and market share relatively low, in quantitative terms. This contrasts with the growing epidemiological profile of cancer in Brazil (27) and with the expectation of new treatments, including precision medicine (28). However, this can be explained by the logic of oncology policy in Brazil, in which antineoplastic drugs are acquired in a decentralized manner by oncology services, with reimbursement values often considered limiting. In this sense, the National Policy for Cancer Prevention and Control in SUS (Law No. 14.758/2023) defines preference for centralized acquisition by the Ministry of Health in cases of high complexity, or high financial impact (29). Based on this new access logic, there may be a change in the incorporation profile for these drugs in SUS.

In terms of compliance with BIA guidelines, the cost of the treatments evaluated represented relevant concerns. While it is recommended to include all direct costs of the technologies compared (such as hospitalization for application and adverse events) (9), costs related to drug administration and management of adverse events were only lightly considered in BIAs. Despite being presented as an optional item in the guidelines, avoided costs were also little explored in BIAs. Furthermore, failures in reporting the quantification of inputs potentially compromise the transparency and reproducibility of BIAs. They also limit support for decision-making on the incorporation of drugs into SUS (30). 

This suggests the need to review the guidelines if the Ministry of Health no longer advocates these recommendations. Otherwise, administrative and legal measures must be taken to ensure that incorporation proposals take into account such recommendations. Furthermore, it is pertinent that the Ministry of Health reflect on cases in which BIA requirement can be relaxed, such as the inclusion of new pharmaceutical presentations in SUS. Thus, the aim is to adequately inform decision-makers about the budgetary viability of incorporating drugs into SUS.

This study systematized the methodological decisions for BIA regarding drugs incorporated into the SUS between 2012 and 2024. The findings indicate that methodological inconsistencies persist in BIA, such as the absence of sensitivity analyses and cost analyses limited to the acquisition cost of drugs. Therefore, it is suggested that there may be weaknesses in the estimates of the real budget impacts of technologies on SUS.

## Data Availability

The database and analysis codes used in this study are available at https://doi.org/10.17632/ytybvjj3br.1.
