# Supplementary material for: Drugs incorporated into the Brazilian Unified Health System: document analysis of methodological decisions in budget impact analyses between 2012 and 2024
Source: Epidemiol Serv Saude. 2025 Sep 8;34:e20250122. doi: 10.1590/S2237-96222025v34e20250122.en (PMC12435967; doi:10.1590/S2237-96222025v34e20250122.en)
Supplement: Translation

# Translation: Medicamentos incorporados no Sistema Único de Saúde: análise documental das decisões metodológicas em análises de impacto orçamentário entre 2012 e 2024

**DOI:** 10.1590/S2237-96222025v34e20250122.pt

**Authors:** Daniel da Silva Pereira Curado, Everton Nunes da Silva

**Affiliations:** 1 Universidade de Brasília, Programa de Pós-Graduação em Saúde Coletiva, Brasília, DF, Brasil; 2 Ministério da Saúde, Secretaria-Executiva da Comissão Nacional de Incorporação de Tecnologias no Sistema Único de Saúde, Brasília, DF, Brasil; 3 Universidade de Brasília, Faculdade de Ciências e Tecnologias em Saúde, Brasília, DF, Brasil

**Keywords:** Análise de Impacto Orçamentário, Avaliação de Tecnologias de Saúde, Medicamentos, Sistema Único de Saúde, Análise Documental

## Abstract

**Objetivo:**

Sistematizar as decisões metodológicas adotadas nos estudos de impacto orçamentário dos relatórios de recomendação da Comissão Nacional de Incorporação de Tecnologias no Sistema Único de Saúde (Conitec) referentes a medicamentos incorporados no SUS no período de 2012 a 2024.

**Métodos:**

Trata-se de estudo exploratório, descritivo, retrospectivo, por meio de análise documental de relatórios técnicos de recomendação da Conitec com decisões de incorporação de medicamentos publicadas até 2024. Informações das Análises de Impacto Orçamentário (AIO) foram extraídas e apresentadas por porcentagem, mediana e intervalo interquartil.

**Resultados:**

Foram identificadas 291 análises para incorporação de medicamentos. Mais da metade destas teve como demandante o setor público (65,3%). As doenças crônicas não transmissíveis foram mais frequentes (39,2%), seguidas de infecciosas (22,3%) e raras (21,0%). Majoritariamente, os medicamentos incluídos representaram novas incorporações (55,3%). A presença de AIO nas análises foi bastante gradual, sendo 100% apenas a partir de 2019. Observaram-se 64,5% de adoção de demanda aferida e 84,0% de análises de sensibilidade no total das AIOs. A mediana total da difusão de incorporação (market-share) variou de 30,0% (1º ano) a 72,0% (5º ano). As análises de custos foram centradas essencialmente no custo de aquisição dos medicamentos. Entretanto, a quantidade de unidades farmacêuticas não foi claramente relatada em 55,5% das análises.

**Conclusão:**

Os achados sinalizam que persistem inconsistências metodológicas nas AIOs, tais como ausência de análises de sensibilidade e análises de custos limitadas aos valores de aquisição dos medicamentos. Portanto, sugere-se que pode haver fragilidades nas estimativas dos reais impactos orçamentários das tecnologias no SUS.

**Aspectos éticos**

Esta pesquisa utilizou bancos de dados de domínio público e anonimizados.

## Introdução

A adoção da Avaliação de Tecnologias em Saúde (ATS) tem sido crescente no mundo, especialmente em sistemas universais de saúde. Nesse contexto, uma avaliação econômica abrangente é essencial para informar a tomada de decisão sobre a potencial incorporação de uma tecnologia (1,2). 

Tendo em vista que uma tecnologia pode ser considerada custo-efetiva, mas não economicamente acessível em termos populacionais, a Análise de Impacto Orçamentário (AIO) é essencial para complementar a Análise de Custo-Efetividade (ACE) (3-5). No caso da ACE, uma medida de eficiência alocativa é fornecida ao se ponderar custos e benefícios clínicos. A AIO, por sua vez, estima o impacto nos recursos financeiros em saúde no curto/médio prazo, o que informa a viabilidade orçamentária (affordability) aos tomadores de decisão (1,6,7). 

Diretrizes metodológicas para elaboração de AIO têm sido estabelecidas globalmente, abrangendo o Brasil (8). Nas diretrizes brasileiras, há recomendações sobre várias etapas metodológicas, incluindo a doença, novo tratamento em avaliação e comparadores, perspectiva da análise, horizonte temporal, estimativa da população-alvo, utilização de recursos (custos), difusão e velocidade da incorporação (market-share) e cenários propostos (9). Entretanto, fragilidades na condução de AIO continuam a ser observadas em termos mundiais (10) e nacionais (11,12).

Em geral, as publicações sobre os relatórios da Comissão Nacional de Incorporação de Tecnologias no Sistema Único de Saúde (Conitec) se restringem à presença ou não de AIO nas propostas de incorporação (11,13,14). Faz-se necessário, portanto, o aprofundamento nas características metodológicas desses estudos, para além das informações sobre as recomendações de incorporação de medicamentos no Sistema Único de Saúde (SUS) (13). Assim, proporciona-se uma visão geral e atual da aplicação das recomendações das diretrizes de AIO do Brasil, bem como a constatação de eventuais aprimoramentos ao longo dos anos.

O objetivo do estudo é sistematizar as decisões metodológicas adotadas nos estudos de impacto orçamentário dos relatórios de recomendação da Conitec referentes a medicamentos incorporados no SUS no período de 2012 a 2024.

## Métodos

### Contexto do estudo

Por meio da Lei nº 12.401/2011, a Conitec foi criada para assessorar o Ministério da Saúde sobre incorporação de tecnologias em saúde no SUS (15,16). Assim, houve uma institucionalização legal da ATS no país (17). Segundo o Decreto nº 7.646/2011, as propostas submetidas à Conitec devem contemplar AIO das tecnologias avaliadas, dentre outros requisitos. Os relatórios técnicos subsidiam as recomendações da Conitec, nas quais são consideradas: i) evidências clínicas da tecnologia; ii) avaliação econômica comparativa dos benefícios e dos custos; iii) impacto orçamentário da incorporação da tecnologia no SUS. As principais recomendações sobre elaboração de AIO estão sumarizadas na Tabela 1.

**Tabela 1:** Etapas metodológicas e principais recomendações das diretrizes metodológicas de análise de impacto orçamentário

Definições da Análise de Impacto Orçamentário	Principais recomendações
**População elegível à tecnologia proposta**	Deve-se: i) estabelecer qual será, exatamente, a indicação do novo tratamento no sistema de saúde, priorizando os pacientes que terão maior benefício terapêutico e/ou eficiência alocativa, de acordo com a ACE; ii) estimar a proporção dos pacientes que se enquadram na indicação estabelecida.
**Tecnologia proposta**	Deve contemplar a tecnologia que está sendo proposta.
**Perspectiva da análise**	Definição de uma das seguintes perspectivas: Sistema Único de Saúde (SUS), em suas diferentes esferas; Sistema de saúde suplementar (planos e convênios de saúde privados); Perspectivas locais do sistema de saúde, como hospitais e centros de saúde.
**Horizonte temporal**	Período de um a cinco anos, com as estimativas de impacto orçamentário relatadas ano a ano. Período mais longo para as tecnologias cuja estabilização de demanda no mercado exija mais tempo.
**Abordagem populacional**	Método epidemiológico: dados de prevalência e incidência da condição clínica de interesse, considerando especificamente seu uso do sistema de saúde adotado na perspectiva; Método da demanda aferida: i) contagem de pacientes cadastrados, quando existe algum tipo de sistema de cadastro específico ou; ii) número histórico, ou de um determinado ano, de pedidos de reembolso por um tratamento médico em um plano de saúde específico.
**Cenário de referência**	Atual padrão de uso dos tratamentos disponíveis para a condição clínica na perspectiva adotada. Os tratamentos atuais devem ser descritos considerando-se os mesmos itens usados para a nova intervenção.
**Cenários alternativos**	Recomenda-se que um a três cenários alternativos sejam simulados. Cada cenário alternativo deverá ser concebido com base nas projeções das quotas de mercado e da taxa de incorporação da tecnologia avaliada.
**Market-share do cenário de referência**	Prefere-se informação procedente de órgãos oficiais ou de publicações científicas sobre o mercado nacional. Alternativamente, podem ser consideradas estimativas de quotas de mercado (atual e projeções) de outros países. O uso do painel de especialistas (por meio do método Delphi, por exemplo) pode ser empregado na caracterização do mercado atual quando outras fontes de dados não estiverem disponíveis.
**Market-share dos cenários alternativos**	Devem ser incluídos na projeção das taxas de difusão: a disponibilidade de equipamento, o treinamento e a capacitação de pessoal, a operação de todo sistema, além de hábitos dos médicos e pacientes. Deve-se considerar que algumas terapias são rapidamente incorporadas, como pelas facilidades, benefícios e pressão de mercado, enquanto outras têm sua incorporação retardada, podendo não chegar a 100%.
**Custos do tratamento**	Todos os custos considerados devem refletir a perspectiva do gestor do orçamento da saúde a quem o estudo se destina, isto é, o valor que, de fato, será desembolsado pelo gestor após a incorporação em questão. Somente os custos diretos devem ser considerados, o que inclui os custos da nova tecnologia em si e aqueles diretamente associados ao seu uso, como aqueles relacionados a internações para aplicação, intervenções adjuvantes e medicamentos usados para prevenir ou tratar eventos adversos. Esse custo combinado “pacote terapêutico” deve ser calculado para a nova tecnologia proposta, em seus diferentes cenários alternativos, e para as outras intervenções que compõem o cenário de referência.
**Custos evitados**	Podem ser estimados custos a serem evitados em decorrência de um benefício clínico da nova tecnologia, como pela redução da necessidade de hospitalizações, cirurgias, hemodiálise ou determinados medicamentos.
**Inflação e taxa de descontos**	Esses ajustes não são recomendados devido ao horizonte temporal curto e pelo fato de o valor da AIO corresponder a um valor presente usado nas estimativas orçamentárias do gestor.
**Análise de sensibilidade**	Pela praticidade na execução e pela fácil interpretação dos resultados, recomenda-se a análise de sensibilidade por cenários.

Conteúdo adaptado das Diretrizes Metodológicas de AIO (9).

### Desenho do estudo 

Trata-se de estudo exploratório, descritivo, retrospectivo, por meio de análise documental de relatórios técnicos de recomendação da Conitec sobre incorporação de medicamentos entre 2012 e 2024. A unidade de análise foram relatórios da Conitec de medicamentos incorporados no SUS. Destes, foram extraídas informações sobre as AIOs e variáveis que caracterizam as demandas. Por serem dados secundários, a ferramenta RECORD (18) foi utilizada para orientar o relato do estudo.

Relatórios técnicos foram selecionados por serem documentos que registram o rito processual de incorporação no SUS, embasando o ato decisório do Ministério da Saúde. Além disso, as demandas por iniciativa do setor público têm suas AIOs publicamente apresentadas apenas nos seus respectivos relatórios técnicos, assim como recálculos feitos por pareceristas em análises críticas a demandas do setor privado. 

Os medicamentos foram as tecnologias de interesse neste estudo por representarem grande parte das demandas de incorporação no SUS. Também são um dos itens que mais impacta no orçamento familiar dos brasileiros (19), principalmente em domicílios de baixa renda (20). Além disso, levantamentos até 2016 apontaram que a maioria das avaliações da Conitec se referiu a medicamentos (11,13). Inclusive, esse padrão se acentuou nos últimos anos, segundo painel de acompanhamento do sítio eletrônico da Conitec, o que aponta para uma relevante predominância de medicamentos no processo de incorporação no SUS.

### Amostra 

Os critérios de inclusão foram: i) ser demanda de incorporação de medicamento; ii) ter tido recomendação de incorporação ou ampliação de uso no SUS; e iii) ter tido decisão publicizada entre 1º de janeiro de 2012 e 31 de dezembro de 2024. O critério de exclusão foi ser reavaliação de medicamento que não alterou a decisão anterior.

### Variáveis e fontes de dados

A fonte de dados foi o sítio eletrônico da Conitec, em que se encontram publicamente todos os relatórios de recomendação das tecnologias analisadas (https://www.gov.br/conitec/pt-br/assuntos/avaliacao-de-tecnologias-em-saude/recomendacoes-conitec). Quando houve preocupações quanto ao relato dos relatórios sobre demandas do setor privado, foram complementarmente consultados os dossiês dos demandantes (disponíveis apenas a partir de 2019), que ficam públicos durante o período de consulta pública do tema. Quando necessário, também foram verificadas as gravações das reuniões da Conitec (disponíveis apenas a partir de 2020).

Foram extraídos dados sobre nome do medicamento; condição clínica; demandante, ano de decisão da incorporação; tipo de abordagem populacional; quantitativo da população elegível no 5º ano; percentuais de market-share no 1º e 5º ano; quantidade de comparadores ativos; e componentes de custos considerados na AIO. O banco de dados construído, com as fórmulas utilizadas, está disponível no repositório Mendeley Data (21). As variáveis adotadas e suas descrições são detalhadas na Tabela 2.

**Tabela 2:** Variáveis definidas para categorização das demandas de medicamentos incorporados no Sistema Único de Saúde

Variável	Descrição
**Tipo de demandante**	Variável categórica com as classificações abaixo.
Público	Propostas de incorporação demandadas apenas pelo Ministério da Saúde e demais órgãos da administração pública ou tanto pelo setor público quanto por instituições privadas.
Privado	Propostas de incorporação demandadas exclusivamente por instituições privadas.
**Tipo de incorporação**	Variável categórica com as classificações abaixo e definida por meio de consulta à Relação Nacional de Medicamentos Essenciais (Rename) e aos próprios relatórios da Comissão Nacional de Incorporação de Tecnologias no Sistema Único de Saúde (Conitec).
Nova incorporação	Medicamento sem registro oficial claro de ter sido incorporado anteriormente para a condição clínica em análise ou para outra no SUS.
Ampliação de uso	Medicamento já incorporado para outra doença ou para a mesma doença, mas em outro subgrupo no SUS. Por exemplo, doença grave (1ª incorporação) e moderada (2ª incorporação).
Nova apresentação	Medicamento já incorporado para a indicação em análise, mas em apresentação farmacêutica distinta daquela proposta. Por exemplo, frasco-ampola (intravenosa) já presente no SUS e foi avaliada a inclusão do comprimido (oral) para a mesma indicação terapêutica.
**Tipo de condição clínica**	Variável categórica com as classificações abaixo e definida com base na descrição presente em cada relatório técnico. Em caso de dúvidas, foram consultados os Protocolos Clínicos e Diretrizes Terapêuticas (PCDT) disponíveis. Considerou-se a condição clínica na qual o medicamento atuaria especificamente. Por exemplo, antibiótico para certa infecção bacteriana em um subgrupo de pacientes com uma doença rara levaria à classificação de doença infecciosa.
Prevenível	Condições clínicas preveníveis por meio de medicamentos profiláticos, como vacinas e contraceptivos.
Infecciosa	Doenças infecciosas, exceto as contempladas em preveníveis.
Rara	Doenças com prevalência de até 65 pessoas em cada 100 mil indivíduos, de acordo com a Política Nacional de Atenção Integral às Pessoas com Doenças Raras do Ministério da Saúde (Portaria GM/MS nº 199/2014). Se necessário, foi consultada a base de dados Orphanet (https://www.orpha.net/), dedicada a doenças raras.
Oncológica	Doenças classificadas como cânceres e síndromes tratadas na oncologia.
Doença crônica não transmissível – DCNT	Condições clínicas crônicas não transmissíveis, exceto as contempladas em raras e oncológicas.
**Presença de Análise de Impacto Orçamentário** (AIO)	Variável dicotômica para indicar o que se considerou uma AIO minimamente conduzida ou relatada. Definiu-se como presença de AIO aqueles relatórios que apresentaram, no mínimo, os valores de impacto orçamentário durante o horizonte temporal adotado.
**Tipo de abordagem populacional**	Variável categórica com as classificações abaixo.
Epidemiológica	Abordagem descrita no relatório como exclusivamente epidemiológica, como a utilização de dados de prevalência e incidência advindos de publicações ou de estimativas populacionais.
Demanda aferida	Adoção de dados de demanda aferida, em conjunto ou não com epidemiológicos. Para definir demanda aferida, considerou-se a expressa descrição no relatório ou a citação sobre uso de bases de dados do DATASUS ou registros nacionais de dados coletados naquela população.
**Nº de comparadores ativos**	Quantidade de comparadores farmacologicamente ativos (outros medicamentos) considerados na AIO, de maneira distinta. Isto é, sem serem agrupados em uma proxy de tratamento.
**Ausência de alternativa terapêutica**	Variável dicotômica sobre a não utilização de um (ou mais) comparador farmacologicamente ativo (outro medicamento) com indicação específica para a condição clínica na AIO.
**População estimada**	Quantitativo da população elegível ao medicamento proposto no último ano do horizonte temporal. Assim, difere-se da população em uso, pois se tratou da quantidade total de pessoas sem qualquer estrato, como a aplicação dos percentuais de market-share.
Market-share	Percentuais de difusão do medicamento proposto no 1º e no 5º ano da AIO, com variações entre 0% e 100%, definidos no relatório.
**Análise de sensibilidade**	Variável dicotômica sobre a realização de análise de sensibilidade, por meio de diagrama de tornado ou análise de cenários. Para esta, considerou-se a apresentação de ao menos um cenário adicional.
**Componentes de custos**	Variável categórica, definida como “sim”, “não especificado” ou “não”, e subdividida entre “identificação”, “mensuração” e “valoração”. A primeira se refere à citação da adoção do componente de custo na análise. A segunda faz referência ao relato do quantitativo específico de unidades consideradas (como x comprimidos ao ano). E a última se tratou da apresentação do valor monetário atribuído à unidade do componente de custo (como o preço do medicamento).
Custos de aquisição dos medicamentos	Custos relacionados exclusivamente à aquisição dos medicamentos. Para a mensuração, considerou-se como “não especificado” se o relatório relatou apenas a posologia utilizada.
Custos de administração dos medicamentos	Custos referentes ao explícito procedimento de administração dos medicamentos. Na mensuração, julgou-se que a quantidade estava implícita caso fosse informada a quantidade anual de utilização do medicamento.
Custos de consultas/visitas de profissionais de saúde	Custos de atendimentos clínicos realizados por médicos e demais profissionais de saúde, incluindo hospitalização/internação e outros procedimentos clínicos conduzidos pelos profissionais que fossem contáveis e descritos no relatório.
Exames de monitoramento e acompanhamento	Custos de testes/exames clínicos ou laboratoriais descritos como procedimentos para monitoramento durante o tratamento em que o medicamento proposto será utilizado.
Manejo dos eventos adversos	Custos de procedimentos com a finalidade exclusiva de identificar e manejar eventos adversos, podendo contemplar exames laboratoriais de indicadores bioquímicos específicos e a utilização de medicamentos para manejar efeitos indesejados do medicamento proposto.
Outros	Custos relacionados a procedimentos diversos menos frequentes, não contemplados anteriormente, como cirurgia específica, transplante ou teste diagnóstico prévio ao tratamento.

Destaca-se que classificação de demandas do setor público (Tabela 2) contemplou aquelas conjuntamente advindas de instituições privadas porque, em relatórios que também tiveram a administração pública como demandante, frequentemente os membros da Conitec consideraram mais adequados os cálculos conduzidos pelos pareceristas, no âmbito da demanda chamada interna.

Apesar de grande parte das condições preveníveis compor as doenças infecciosas e de raras e oncológicas também serem doenças crônicas não transmissíveis (DCNT), optou-se por essa desagregação para contemplar as especificidades dessas condições e seus medicamentos. As tecnologias profiláticas (principalmente vacinas e contraceptivos) comumente têm esquemas posológicos diferenciados e têm uma população elegível maior. Isto se deve ao fato de essas pessoas ainda estarem suscetíveis à doença a ser prevenida, o que configura um propósito distinto do tratamento de doenças infecciosas como infecção pelo HIV ou hepatite C. No caso das DCNT, as doenças raras possuem um quantitativo populacional menor, mas uma demanda maior por alternativas terapêuticas, o que frequentemente reflete em percentuais de market-share maiores. As doenças oncológicas, por sua vez, não só estão acompanhadas de custos consideráveis, mas também de uma política de financiamento a medicamentos distinta daquela adotada para as DCNT em geral (política oncológica e política de assistência farmacêutica, respectivamente).

A abordagem populacional adotada na AIO, por sua vez, teve a classificação de demanda aferida agrupada (Tabela 2) para referir análises que essencialmente se apoiaram em dados de demanda aferida, mesmo que tenham utilizado parcialmente dados epidemiológicos. Isto é, quando a base populacional do cálculo foi o quantitativo atual de usuários do SUS, por exemplo. Esta escolha metodológica foi feita para refletir melhor a magnitude da população estimada em cada uma das abordagens, comumente sendo numericamente superior na epidemiológica (9). Dessa forma, AIO que utilizaram tanto a abordagem epidemiológica quanto a demanda aferida tendem a obter estimativas mais próximas das AIO apoiada apenas na demanda aferida do que daquelas com a epidemiológica exclusivamente.

Comparadores que fizeram referência a “tratamento padrão”, “cuidados de suporte”, “cuidados paliativos” ou termos correlatos que não contemplassem explicitamente medicamento com atuação específica naquela doença foram considerados “ausência de comparador ativo”. Ademais, apenas comparadores ativos que foram claramente apresentados na AIO (por exemplo, com market-share e/ou valores distintos) foram contabilizados. Caso tenha sido utilizada uma proxy de medicamentos utilizados, contabilizou-se como apenas um comparador ativo.

Devido à apresentação de diversos cenários nas AIOs, definiu-se aquele que fosse mais representativo da incorporação, do qual foram retirados os dados. Para isso, consideraram-se os seguintes critérios nesta ordem: i) recomendação final da Conitec, definindo população e subgrupos contemplados; ii) recálculo após novas informações, como solicitações da Conitec por ajustes ou novas propostas de preços enviadas pelas empresas; iii) posição explícita dos pareceristas sobre o cenário mais adequado, apresentado à Conitec como principal resultado; iv) difusão mais gradual, indicando implementação mais factível no SUS; v) utilização de dados de demanda aferida, tendendo a ser mais fidedigna à população elegível. Ressalta-se que, nos casos de avaliação de mais de um medicamento no mesmo relatório, foram selecionados os cenários em que os medicamentos incorporados eram considerados de maneira complementar. Quando as informações da AIO não estavam desagregadas, classificou-se como uma AIO única, independentemente da quantidade de medicamentos incorporados. 

Tanto o quantitativo da população elegível quanto os percentuais de market-share foram diretamente extraídos do relatório do medicamento. Entretanto, quando houve algum problema de relato com ausência de certos dados, buscou-se obter as informações por cálculo dos demais dados, quando possível. Por exemplo, o quantitativo de pacientes em uso do medicamento e o market-share no 5º ano foram suficientes para obtenção da população elegível no 5º ano.

No caso dos componentes de custos, focou-se na clareza da descrição de cada item, quanto à identificação (listar os itens), mensuração (quantificar) e valoração (atribuir valor) (22). Nesse sentido, com exceção da mensuração da administração dos medicamentos, não foi feita inferência sobre a quantidade de unidades na AIO. Entretanto, classificou-se como “não especificado” quando não houve apresentação da quantidade de um componente apesar de sua inclusão. Essa classificação também foi dada quando o relato demonstrou indícios daquele componente de custos, mas não o detalhou.

### Análise estatística

Os dados foram analisados de forma agregada, sendo apresentados por porcentagem, mediana e intervalo interquartil para representar medida de tendência central e dispersão, respectivamente. 

Para evitar potenciais fontes de viés (18), foram considerados todos os relatórios com dados disponíveis de AIO. Ademais, realizaram-se interpolação e recálculo possíveis de dados faltantes devido a falhas de relato dos relatórios.

Análises de subgrupos foram realizadas para demonstrar diferenças entre os medicamentos. Essas estratificações foram feitas por ano de incorporação e tipo de condição clínica. Os resultados foram apresentados em tabelas, com as devidas estratificações e totais.

## Resultados

No período, 291 análises para incorporação de medicamentos foram identificadas. Mais da metade destas teve como demandante o setor público (65,3%), variando de 44,4% em 2018 a 92,3% em 2014 e 2015 ao longo do período. As DCNT foram mais frequentes (39,2%), seguidas de infecciosas (22,3%) e raras (21,0%). No entanto, esse padrão foi heterogêneo durante os anos avaliados, sem uma tendência bem definida para a condição clínica estratificada por ano (Tabela 3).

**Tabela 3:** Caracterização geral das demandas de medicamentos incorporados no Sistema Único de Saúde entre 2012 e 2024. Brasil, 2025 (n=291)

Ano (n)	Demandante	Condição clínica					Tipo de incorporação		Presença de Análise de Impacto Orçamentário n (%)
	Público n (%)	Prevenível n (%)	Infecciosa n (%)	Rara n (%)	Oncológica n (%)	Doença crônica não transmissível n (%)	Nova incorporação n (%)	Ampliação de uso n (%)	
**2012** (24)	22 (91,7)	1 (4,2)	3 (12,5)	3 (12,5)	2 (8,3)	15 (62,5)	20 (83,3)	4 (16,7)	-
**2013** (15)	12 (80,0)	4 (26,7)	1 (6,7)	3 (20,0)	4 (26,7)	3 (20,0)	12 (80,0)	3 (20,0)	2 (13,3)
**2014** (13)	12 (92,3)	-	2 (15,4)	2 (15,4)	5 (38,5)	4 (30,8)	2 (15,4)	10 (76,9)	3 (23,1)
**2015** (26)	24 (92,3)	-	12 (46,2)	-	1 (3,8)	13 (50,0)	6 (23,1)	14 (53,8)	5 (19,2)
**2016** (12)	7 (58,3)	-	3 (25,0)	-	-	9 (75,0)	3 (25,0)	8 (66,7)	6 (50,0)
**2017** (24)	13 (54,2)	-	6 (25,0)	9 (37,5)	1 (4,2)	8 (33,3)	11 (45,8)	10 (41,7)	16 (66,7)
**2018** (27)	12 (44,4)	-	10 (37,0)	10 (37,0)	2 (7,4)	5 (18,5)	19 (70,4)	4 (14,8)	22 (81,5)
**2019** (19)	11 (57,9)	2 (10,5)	2 (10,5)	3 (15,8)	2 (10,5)	10 (52,6)	11 (57,9)	4 (21,1)	19 (100,0)
**2020** (25)	14 (56,0)	2 (8,0)	6 (24,0)	5 (20,0)	4 (16,0)	8 (32,0)	15 (60,0)	7 (28,0)	22 (88,0)
**2021** (19)	10 (52,6)	2 (10,5)	2 (10,5)	4 (21,1)	3 (15,8)	8 (42,1)	12 (63,2)	3 (15,8)	19 (100,0)
**2022** (35)	20 (57,1)	2 (5,7)	9 (25,7)	10 (28,6)	3 (8,6)	11 (31,4)	20 (57,1)	13 (37,1)	34 (97,1)
**2023** (22)	11 (50,0)	2 (9,1)	4 (18,2)	7 (31,8)	2 (9,1)	7 (31,8)	14 (63,6)	3 (13,6)	22 (100,0)
**2024** (30)	22 (73,3)	-	5 (16,7)	5 (16,7)	7 (23,3)	13 (43,3)	16 (53,3)	14 (46,7)	30 (100,0)
**Total** (291)	**190** (65,3)	**15** (5,2)	**65** (22,3)	**61** (21,0)	**36** (12,4)	**114** (39,2)	**161** (55,3)	**97** (33,3)	**200** (68,7)

Em cada uma das colunas, o gradiente de cor representa o intervalo dos achados. Assim, tons mais escuros da cor indicam percentuais maiores na respectiva coluna.

Majoritariamente, os medicamentos incluídos representaram novas incorporações (55,3%), com percentuais acima de 53,0% durante o período, exceto de 2014 a 2017, quando as ampliações de uso se destacaram. Por outro lado, a incorporação de novas apresentações sempre foi bastante discreta, nunca passando de 24,0%, percentual de 2015 (Tabela 3).

Conforme observado na Tabela 3, a presença de AIO nos relatórios técnicos sobre medicamentos foi bastante gradual nos primeiros anos da Conitec, chegando a 50,0% em 2016. A partir de 2018, os percentuais de AIO se mantiveram acima de 80,0%. Com exceção de 2020 e 2022, em que foram obtidos percentuais de 88,0% e 97,1%, AIO foi contemplada adequadamente na totalidade das análises conduzidas nos últimos anos.

Quanto às escolhas metodológicas das AIO (Tabela 4), as análises de medicamentos para condições preveníveis se destacaram ao ter população estimada maior que as das demais condições clínicas. Esse padrão também foi observado no market-share do 1º e 5º ano das preveníveis (medianas de 75,0% a 100,0%, respectivamente). Em segundo lugar, quanto ao tamanho de população estimada, estiveram as DCNT. Entretanto, as DCNT apresentaram os menores percentuais de market-share (medianas de 26,0% no 1º ano e 60,0% no 5º ano). Em termos de população e market-share, as doenças raras apresentaram um perfil bastante parecido com as oncológicas (população relativamente pequena e market-share moderado).

**Tabela 4:** Características metodológicas adotadas nas análises de impacto orçamentário referentes a medicamentos incorporados no Sistema Único de Saúde entre 2012 e 2024. Brasil, 2025 (n=291)

Tipo de condição clínica	População estimada (n; IQT)^a^	Market share (%; IQT)^a^	Comparadores (n; IQT)^a^	Sem alternativa terapêutica n (%)	Demanda aferida n (%)	Análise de sensibilidade n (%)
Preveníveis	1.887.115 (270.914; 5.323.049)	**1º ano**: 75,0 (19,3; 100,0)	1 (0; 1)	4 (44,4)	4 (44,4)	8 (88,9)
		**5º ano**: 100,0 (21,3; 100,0)				
Infecciosas	3.115 (592; 41.114)	**1º ano**: 50,0 (30,0; 100,0)	1 (1; 1)	8 (20,5)	23 (59,0)	33 (84,6)
		**5º ano**: 95,0 (50,0; 100,0)				
Raras	1.550 (232; 5.296)	**1º ano**: 50,0 (20,0; 65,0)	1 (0; 2)	22 (44,9)	29 (59,2)	40 (81,6)
		**5º ano**: 80,0 (45,0; 95,0)				
Oncológicas	737 (224; 3.849)	**1º ano**: 30,0 (10,0; 87,5)	1 (1; 1)	4 (14,8)	20 (74,1)	20 (74,1)
		**5º ano**: 70,0 (50,0; 100,0)				
**Doenças crônicas não transmissíveis**	28.150 (9.087; 85.747)	**1º ano**: 26,0 (10,0; 50,0)	1 (1; 2)	15 (19,7)	53 (69,7)	67 (88,2)
		**5º ano**: 60,0 (36,2; 100,0)				
Total	5.619 (814; 45.228)	**1º ano**: 30,0 (15,0; 100,0)	1 (0; 1)	53 (26,5)	129 (64,5)	168 (84,0)
		**5º ano**: 72,0 (45,0; 100,0)				

IQT – intervalo interquartil; ^a^Mediana e IQT (1º e 3º quartis, respectivamente).

Em relação à ausência de alternativa terapêutica, isto é, comparador farmacologicamente ativo na AIO, as doenças raras e as preveníveis apresentaram percentuais próximos de 45,0%, enquanto as demais não passaram de 21,0%. Entretanto, ressaltam-se as diferentes quantidades de análises sem alternativa terapêutica entre condições preveníveis (n=4) e raras (n=22) (Tabela 4).

As doenças oncológicas representaram o tipo de condição clínica em que mais frequentemente se adotou demanda aferida na abordagem populacional, sendo 74,1% das AIOs. De todo modo, a grande maioria das condições clínicas demonstrou percentuais acima de 58,0%, exceto as preveníveis (44,4%) (Tabela 4).

Quanto à realização de análises de sensibilidade, as condições preveníveis e DCNT se destacaram com percentuais acima de 88,0%. Enquanto as oncológicas apresentaram percentual menor (74,1%), a realização de análise de sensibilidade no geral das AIOs foi de 84,0% (Tabela 4).

Observa-se, a partir da Tabela 5, que medicamento foi o componente de custo mais contemplado nas AIOs, estando na totalidade das análises. Entretanto, destaca-se que, em mais da metade das vezes (55,5%), a quantidade de unidades não foi claramente relatada. Vale ressaltar que este domínio de mensuração apresentou os menores percentuais em todos os componentes de custo. Contudo, o manejo dos eventos adversos e outros foram os itens menos contemplados em todos os domínios nas AIOs (≈16,0%).

**Tabela 5:** Componentes de custos considerados nas análises de impacto orçamentário referentes a medicamentos incorporados no Sistema Único de Saúde entre 2012 e 2024. Brasil, 2025 (n=200)

Componente de custo	Identificação		Mensuração		Valoração	
	Sim n (%)	Não especificado n (%)	Sim n (%)	Não especificado n (%)	Sim n (%)	Não especificado n (%)
Medicamento	200 (100,0)	0 (0,0)	89 (44,5)	111 (55,5)	198 (99,0)	1 (0,5)
**Administração de medicamento**	31 (15,5)	0 (0,0)	16 (8,0)	15 (7,5)	28 (14,0)	3 (1,5)
**Consultas/visitas de profissionais de saúde**	51 (25,5)	1 (0,5)	19 (9,5)	33 (16,5)	49 (24,5)	3 (1,5)
**Exames de monitoramento e acompanhamento**	46 (23,0)	1 (0,5)	21 (10,5)	26 (13,0)	43 (21,5)	4 (2,0)
**Manejo dos eventos adversos**	31 (15,5)	0 (0,0)	9 (4,5)	21 (10,5)	28 (14,0)	2 (1,0)
Outros	32 (16,0)	0 (0,0)	8 (4,0)	24 (12,0)	27 (13,5)	5 (2,5)

Em cada uma das colunas, o intervalo dos achados é representado pelo gradiente das cores verde (percentuais superiores), amarelo (percentuais medianos) e vermelho (percentuais inferiores). Assim, tons mais escuros de cada cor indicam valores que tendem aos extremos (verde e vermelho) ou ponto central (amarelo) na respectiva coluna.

## Discussão

Houve avanço metodológico das AIOs de medicamentos incorporados no SUS, sobretudo a partir de 2019. Contudo, foram observadas inconsistências na aplicação das diretrizes de AIO, especialmente quanto aos componentes de custos incluídos. Grande parte das análises consideraram apenas os custos dos medicamentos sob investigação, não contemplando demais custos relacionados ao tratamento da condição de saúde. Além disso, não foi verificada a realização de análises de sensibilidade em parte dos relatórios de incorporação, o que implica a ausência de avaliação das incertezas. Esses achados indicam que pode haver fragilidades nas estimativas dos reais impactos orçamentários das tecnologias no SUS.

Este foi o primeiro estudo a sistematizar as características de AIO de relatórios da Conitec, confrontando-as às diretrizes metodológicas de AIO. Para isso, foram propostas definições claras e objetivas. Ademais, foram utilizados exclusivamente dados públicos e oficiais da Conitec. Isso permite a reprodutibilidade e continuidade do monitoramento das AIOs de medicamentos incorporados no SUS. Contudo, algumas limitações presentes neste estudo devem ser pontuadas. Primeiro, algumas informações relevantes para compreensão das AIOs estavam incompletas ou ausentes nos relatórios da Conitec, o que reduziu a amostra da nossa análise e não representou a totalidade das AIOs de incorporações de medicamentos no SUS. Segundo, a definição de “presença de AIO” implicou a não inclusão de determinados relatórios com informações insuficientes de AIO. Justifica-se, entretanto, que a incompletude desses relatórios impediria que tais dados agregassem aos resultados desse estudo e potencialmente aumentariam a heterogeneidade dos achados. Terceiro, o primeiro ano das AIOs não necessariamente corresponde ao ano de incorporação do medicamento, o que pode representar distorções na população estimada. Quarto, os medicamentos e seus comparadores que não foram desagregados nas AIOs foram considerados por nós como apenas um, o que não representa a realidade da prática clínica. Quinto, as informações utilizadas se restringiram a apenas um dos cenários descritos no relatório do medicamento, selecionado pelos critérios que definimos. Esse cenário não necessariamente representa a realidade do medicamento no SUS, que potencialmente envolverá diversas variáveis que desconhecemos. Sexto, algumas informações faltantes da AIO foram calculadas por nós quando as demais relatadas permitiam este procedimento. Justifica-se que, assim, buscou-se mitigar a perda de dados nas análises. Entretanto, reconhece-se a possibilidade de inconsistências nestes dados calculados, apesar de se basearem exclusivamente nas demais informações descritas nos relatórios.

A versão vigente das diretrizes metodológicas de AIO, publicadas em 2012, tem sido disponibilizada para nortear a elaboração de AIO (9). Com a exigência legal de sua observância para propor incorporações no SUS, os demandantes tiveram de se adaptar quanto à apresentação desse tipo de estudo. Levantamento realizado entre 2012 e 2015 revelou que 17,3% das demandas não foram apreciadas pela Conitec por terem sido recusadas na análise documental prévia devido à ausência de AIO especificamente (23). Esse percentual é próximo da média de presença de AIO entre 2012 e 2015 observada no nosso estudo (13,9%). Destaca-se que este foi o período em que os medicamentos incorporados foram majoritariamente demandados pelo setor público, o que pode estar relacionado à avaliação pela Conitec mesmo sem AIO nos termos das diretrizes.

Gradualmente, os demandantes se adaptaram às diretrizes de AIO, chegando a 100% em 2019. Entretanto, em 2020 e 2022, esses percentuais caíram em algumas propostas do setor público para inclusão de nova apresentação farmacêutica – fármacos já disponíveis no SUS, mas em apresentações distintas da proposta. Esses casos reforçam a discussão sobre pertinência da incorporação de novas apresentações farmacêuticas no SUS, no âmbito da Conitec, quando não há preocupações relevantes sobre sua eficácia/efetividade, segurança, custo-efetividade e impacto orçamentário. Ademais, tem-se observado sugestões de “desburocratizar o processo de submissão de propostas de avaliação de tecnologias à Conitec, quando submetidas pelos entes do SUS” em discussões promovidas pelo Ministério da Saúde (24).

Conforme as diretrizes de AIO, o método epidemiológico para estimar a população elegível tende a superestimar o quantitativo real de pessoas (9). Assim, a recomendação das diretrizes sobre preferir por estimativas com demanda aferida pode explicar a majoritária adoção de demanda aferida nas AIOs que analisamos. Adicionalmente, populações menores, obtidas por demanda aferida, resultam em valores menores de impacto orçamentário. Como um importante elemento no processo de tomada de decisão (6), a AIO pode ser decisiva quanto à incorporação do medicamento. Até 2016, 25,8% dos medicamentos incorporados no SUS tiveram baixo impacto financeiro-orçamentário como justificativa no relatório da Conitec (13).

Tendo em vista que dados de mundo real nem sempre estão disponíveis no momento da incorporação do medicamento, iniciativas têm sido executadas pelo Ministério da Saúde para mitigar riscos à sustentabilidade do SUS. Em 2017, diretrizes metodológicas de avaliação de desempenho de tecnologias em saúde, com foco em dados de mundo real, foram publicadas (25). Posteriormente, a Portaria GM/MS nº 4.228/2022 permitiu que as deliberações da Conitec sejam provisórias e condicionadas à reavaliação de parâmetros, incluindo o impacto orçamentário da tecnologia no SUS. Medidas como essas facilitam a revisão de incorporações após serem verificadas relevantes diferenças entre o impacto orçamentário estimado e o real (12).

A elevada adoção de demanda aferida observada neste estudo pode estar relacionada aos percentuais gerais de market-share. Isso contemplaria a recomendação das diretrizes de AIO sobre considerar dados de dispensação ou reembolso de medicamentos como uma das formas para definir o *market-share* (9). Destaca-se que os menores percentuais correspondem aos medicamentos incorporados para DCNT, que majoritariamente exigem a elaboração ou atualização de PCDT da doença para serem disponibilizados no SUS. Esse processo do PCDT, aquisição e dispensação do medicamento para condições prevalentes comumente requerem vários meses após a incorporação em si (26), o que pode ser considerado na definição do market-share. Ademais, a maioria das demandas se referiu às DCNT, que tiveram uma das menores ausências de alternativa terapêutica (19,7%). Isso indica que muitos desses medicamentos foram incorporados como mais uma das opções terapêuticas daquela condição clínica e, consequentemente, passaram a dividir mercado com elas. Tal fato justificaria seu market-share mais baixo, que não atinge 100% após os cinco anos da AIO.

Os medicamentos incorporados para doenças oncológicas, por sua vez, apresentaram um quantitativo populacional e market-share relativamente baixos. Isso contrasta com o perfil epidemiológico crescente dos cânceres no Brasil (27) e com a expectativa de novos tratamentos, inclusive a medicina de precisão (28). Entretanto, isso pode ser explicado pela lógica da política oncológica no Brasil, em que os antineoplásicos são adquiridos de maneira descentralizada pelos serviços de oncologia, com valores de reembolso frequentemente considerados limitantes. Nesse sentido, a Política Nacional de Prevenção e Controle do Câncer no SUS (Lei nº 14.758/2023) define preferência para a aquisição centralizada pelo Ministério da Saúde nos casos de alta complexidade, ou impacto financeiro elevado (29). A partir dessa nova lógica de acesso, poderá haver uma mudança no perfil de incorporação para esses medicamentos no SUS.

Em termos de adequação às diretrizes de AIO, o custeio dos tratamentos avaliados representou relevantes preocupações. Enquanto é recomendada a inclusão de todos os custos diretos das tecnologias comparadas (como de internação para aplicação e de eventos adversos) (9), custos relacionados à administração do medicamento e manejo de eventos adversos foram minoritariamente considerados nas AIOs. Apesar de serem apresentados como um item opcional das diretrizes, os custos evitados também foram pouco explorados nas AIOs. Ademais, as falhas no relato da quantificação dos insumos potencialmente comprometem a transparência e reprodutibilidade das AIOs. Assim também, limitam os subsídios à tomada de decisão sobre a incorporação de medicamento no SUS (30). 

Isso sugere a necessidade de revisão das diretrizes, caso o Ministério da Saúde não mais preconize essas recomendações. Caso contrário, medidas administrativas e legais devem ser tomadas para garantir que as propostas de incorporação contemplem tais recomendações. Ademais, é pertinente que o Ministério da Saúde reflita sobre os casos em que a exigência de AIO pode ser flexibilizada, como inclusão de novas apresentações farmacêuticas no SUS. Assim, busca-se informar adequadamente a viabilidade orçamentária da incorporação de medicamentos no SUS aos tomadores de decisão.

Este estudo sistematizou as decisões metodológicas de AIO referentes a medicamentos incorporados no SUS entre 2012 e 2024. Os achados sinalizam que persistem inconsistências metodológicas nas AIOs, tais como ausência de análises de sensibilidade e análises de custos limitadas aos valores de aquisição dos medicamentos. Portanto, sugere-se que pode haver fragilidades nas estimativas dos reais impactos orçamentários das tecnologias no SUS.

## Data Availability

O banco de dados e os códigos de análise utilizados na pesquisa estão disponíveis em https://doi.org/10.17632/ytybvjj3br.1.
